# Associations between the perception of risk in radiation exposure and changes in smoking and drinking status after a disaster: The Fukushima Health Management Survey

**DOI:** 10.1016/j.pmedr.2022.102054

**Published:** 2022-11-14

**Authors:** Tomohiko Ukai, Takahiro Tabuchi, Tetsuya Ohira, Hironori Nakano, Masaharu Maeda, Hirooki Yabe, Atsushi Takahashi, Seiji Yasumura, Hiroyasu Iso, Kenji Kamiya

**Affiliations:** a Division of Public Health, Osaka Institute of Public Health, 1-3-69 Nakamichi, Higashinari-ku, Osaka, Osaka 537-0025, Japan; b Public Health, Department of Social Medicine, Osaka University Graduate School of Medicine, 2-2 Yamadaoka, Suita, Osaka 565-0871, Japan; c Cancer Control Center, Osaka International Cancer Institute, 3-1-69 Otemae, Chuo-ku, Osaka 541-8567, Japan; d Radiation Medical Science Center for the Fukushima Health Management Survey, Fukushima Medical University, Hikarigaoka 1, Fukushima, Fukushima 960-1205, Japan; e Department of Epidemiology, Fukushima Medical University School of Medicine, Hikarigaoka 1, Fukushima, Fukushima 960-1205, Japan; f Department of Disaster Psychiatry, Fukushima Medical University School of Medicine, Hikarigaoka 1, Fukushima, Fukushima 960-1205, Japan; g Department of Neuropsychiatry, Fukushima Medical University School of Medicine, Hikarigaoka 1, Fukushima, Fukushima 960-1205, Japan; h Department of Gastroenterology, Fukushima Medical University School of Medicine, Hikarigaoka 1, Fukushima, Fukushima 960-1205, Japan; i Department of Public Health, Fukushima Medical University School of Medicine, Hikarigaoka 1, Fukushima, Fukushima 960-1205, Japan; j Department of Public Health, Faculty of Medicine, University of Tsukuba, 1-1-1 Tennodai, Tsukuba, Ibaraki 305-8575, Japan; k Research Institute for Radiation Biology and Medicine, Hiroshima University, Hiroshima, Japan

**Keywords:** Natural disaster, Smoking, Alcohol consumption, Mental distress, Radiation exposure

## Abstract

The risk for people evacuated from Fukushima following the Great East Japan Earthquake of developing cancer from radiation exposure may be lower than that associated with smoking and alcohol drinking. However, the perception of those risks may change risk-related behavior. Therefore, we investigated whether the perceived risk of radiation exposure was associated with the initiation and/or cessation of smoking and of drinking alcohol following the disaster.

Participants were 82,197 people aged ≥20 years who completed the Fukushima Health Management Study survey. A multivariable logistic regression model, with adjusted odds ratios (AORs) and 95 % confidence intervals (CIs), was used to calculate the risk of (1) starting smoking (or drinking) among people who did not smoke (or drink) before the earthquake, and (2) quitting smoking (or drinking) among people who smoked (or drank) before the earthquake; the main factor was perceived risk of developing cancer from radiation.

The AORs for starting smoking among participants who perceived radiation exposure risks as unlikely, likely, and very likely, compared with very unlikely, were 0.96(0.78–1.18), 1.17(0.95–1.45), and 1.69(1.39–2.06), respectively (Trend p < 0.01). The corresponding ORs for starting drinking were 1.05 (0.95–1.16), 1.17(1.06–01.30), and 1.38(1.25–1.52), respectively (Trend p < 0.01). The AORs for quitting smoking were 0.90(0.82–0.98), 0.81(0.73–0.90), and 0.75(0.68–0.83), respectively (Trend p < 0.01). The same association was not found among alcohol quitters.

In Fukushima, people who perceived greater risk of developing cancer from radiation exposure had higher odds of starting smoking and drinking alcohol, which, ironically, increases the risk of developing cancer.

## 1 Introduction

The Great East Japan Earthquake was one of the world’s most devastating natural disasters (Nakano et al., 2018). In Fukushima, more than 160,000 people were evacuated because of the Fukushima Daiichi nuclear power plant explosion that followed the earthquake and the tsunami (Nakano et al., 2018). Although they avoided ongoing radiation exposure, residents did receive some radiation exposure before they were evacuated. Studies of atomic bombs and nuclear plant accidents suggest that radiation exposure is associated with increased risk of developing cancer such as bladder and thyroid cancer, and increased risk of heart diseases owing to ionizing radiation (Kamiya et al., 2015, Hasegawa et al., 2015).

However, the effects of the radiation exposure that the people in Fukushima experienced was reportedly low. (WHO, 2011) A few researchers reported that the radiation exposure increased the instances of thyroid cancer (Tsuda et al., 2016) or congenital heart disease (Murase et al., 2019), but other researchers rejected these claims (Ohtsuru et al., 2019, Ohira et al., 2016). Furthermore, The United Nations Scientific Committee on the Effects of Atomic Radiation stated that the dose to which the public was exposed was estimated to be low and the health effects are thought to be small (United Nations Scientific Committee on the Effects of Atomic Radiation).

While the radiation risks in this situation are expected to remain low, Fukushima residents’ perceptions of the risks of radiation may have affected their health behaviors. As previous studies have shown, a disaster can change the behaviors of the affected people (Keyes et al., 2011, Vlahov et al., 2004, Vlahov et al., 2002). Evacuated people may experience tremendous stress from fear of radiation exposure, and therefore, it is possible that people in Fukushima may have changed their health-related behaviors, such as taking up smoking or drinking alcohol, in response to that stress. These behavior changes are not desirable because drinking alcohol and smoking are known risk factors for cancer (Yabe et al., 2014, Nelson et al., 2013). Arguably, these people need support to maintain or adopt healthy lifestyles. The research question in the present study, then, was whether perceived radiation exposure risk was associated with the initiation and/or cessation of smoking and of alcohol drinking after the Great East Japan Earthquake.

## 2 Methods

### 2.1 Participants

Following the Great East Japan Earthquake that occurred on March 11, 2011, the Japanese government created evacuation instruction zones in the affected areas. Beginning in 2012, many evacuees participated in the Fukushima Health Management Survey, which has been delivered annually between January and October and addresses the Fukushima Daiichi nuclear power plant accident that was precipitated by the earthquake. Part of the above longitudinal study includes the Mental Health and Lifestyle Survey, which assesses how the disaster and evacuees’ lifestyles have affected their mental status over a long period of time (Yabe et al., 2014, Yasumura et al., 2012).

The target population for the survey comprised men and women aged 15 years or older who lived in the following evacuation zones specified by the government: Hirano town, Naraha town, Futaba town, Namie town, Katsurao village, Minamisoma city, Tamura city, Kawamata town, Iitate village, and a part of Date city. The survey was administered from 30 January to 31 October 2012. The questionnaire was mailed to people who possessed a certificate of residence in the evacuation area as of March 11, 2011. The research experts guaranteed precision when recording the data, and we double-checked all entered data.

## 3 Variables

### 3.1 Outcome variables

Because our interest was in changes in smoking and alcohol-drinking behavior among evacuees, we limited participants to people who were at least 20 years old, which is the legal smoking and drinking age in Japan. Consequently, 74,667 participants’ data were used for analysis.

The questionnaire was used to determine participants’ smoking status before the disaster, which was classified as “current smoker” or “current non-smoker.” Current smoking status (i.e., when answering the questionnaire) was classified as “never smoked,” “ex-smoker,” or “current smoker.” Changes in smoking status—from before to after the disaster—were categorized into four groups as shown in Table 1: (1) non-smoker both before and after the disaster (non-smokers); (2) non-smoker before and smoker after the disaster (starters); (3) smoker before and non-smoker after the disaster (quitters); and (4) smoker both before and after the disaster (smokers). Similarly, changes in drinking status before and after the disaster were categorized into four groups: (1) non-drinker both before and after the disaster (non-drinkers); (2) non-drinker before and drinker after the disaster (starters); (3) drinker before and non-drinker after the disaster (quitters); and (4) drinker both before and after the disaster (drinkers).

**Table 1:** Baseline characteristics according to the perception of radiation exposure risk (N = 74,667).

	Perception of radiation exposure risk							
	Very unlikely (n = 20 135)		Unlikely (n = 24 181)		Likely (n = 18 197)		Very likely (n = 19 684)	
Demographic characteristics	n	%	n	%	n	%	n	%
Sex (female)	9689	(48.1)	13,218	(54.7)	10,628	(58.4)	11,280	(57.3)
Age								
20–49	5567	(27.6)	8502	(35.2)	6885	(37.8)	7097	(36.1)
50–64	6652	(33.0)	7831	(32.4)	5296	(29.1)	5806	(29.5)
≥65	8016	(39.8)	7848	(32.5)	6016	(33.1)	6781	(34.4)
Education								
Primary or junior high school	5510	(27.4)	5584	(23.1)	4359	(24.0)	4997	(25.4)
High school	8967	(44.5)	11,550	(47.8)	8695	(47.8)	9390	(47.7)
Two-year college	2855	(14.2)	4160	(17.2)	3200	(17.6)	3205	(16.3)
University or postgraduate school	2233	(11.1)	2303	(9.5)	1389	(7.6)	1299	(6.6)
Missing data	570	(2.8)	584	(2.4)	554	(3.0)	793	(4.0)
Smoking status								
Never smoked	10,288	(51.1)	12,970	(53.6)	9694	(53.3)	9797	(49.8)
Past smoker	4902	(24.3)	5204	(21.5)	3791	(20.8)	4133	(21.0)
Current smoker	4045	(20.1)	5204	(21.5)	3942	(21.7)	4721	(24.0)
Drinking status								
Never drank	9454	(47.0)	11,925	(49.3)	8996	(49.4)	9577	(48.7)
Past drinker	784	(3.9)	763	(3.2)	638	(3.5)	732	(3.7)
Current drinker	9356	(46.5)	11,065	(45.8)	8155	(44.8)	8754	(44.5)
Physical exercise								
Almost everyday	3175	(15.8)	3041	(12.6)	2225	(12.2)	2758	(14.0)
2–4 /week	3862	(19.2)	4504	(18.6)	3567	(19.6)	3543	(18.0)
1 /week	2796	(13.9)	3494	(14.4)	2500	(13.7)	2597	(13.2)
Rarely	9837	(48.9)	12,683	(52.5)	9536	(52.4)	10,323	(52.4)
Missing data	465	(2.3)	459	(1.9)	369	(2.0)	463	(2.4)
Past medical history								
Hypertension	8737	(43.4)	9513	(39.3)	7217	(39.7)	8367	(42.5)
Diabetes	3842	(19.1)	4109	(17.0)	3261	(17.9)	4113	(20.9)
Hypercholesterolemia	6662	(33.1)	8021	(33.2)	6164	(33.9)	7141	(36.3)
Stroke	1091	(5.4)	1003	(4.1)	814	(4.5)	1061	(5.4)
Heart disease	1931	(9.6)	2113	(8.7)	1763	(9.7)	2135	(10.8)
Cancer	934	(4.6)	1105	(4.6)	859	(4.7)	1017	(5.2)
Mental illness	1129	(5.6)	1243	(5.1)	1190	(6.5)	1690	(8.6)

### 3.2 Exposure variables

The perception of radiation exposure risk was assessed with the following question: “What do you think is the likelihood of damage to your health (e.g., cancer onset) in later life as a result of your current level of radiation exposure?” The question was translated into Japanese, then back to English. Participants were asked to respond to each question using a four-point Likert scale: “very unlikely,” “unlikely,” “likely,” or “very likely.”

### 3.3 Potential confounders

Potential confounders included age (20–49 years old, 50–64, and ≥ 65), sex, educational attainment (elementary school or junior high school, high school, vocational college or junior college, and university or graduate school), physical exercise (almost every day, 2–4 times/week, 1 time/week, or rarely), medical histories of hypertension (yes or no), diabetes (yes or no), hyperlipidemia (yes or no), stroke (yes or no), ischemic heart disease (yes or no), cancer (yes or no), and mental illness (yes or no). Smoking and drinking status were mutually adjusted.

### 3.4 Statistical analysis

The mean values and prevalence of selected factors were calculated based on the recognition of the risks of radiation exposure, and the overall difference across groups was tested using analysis of covariance.

The percentage of missing values across the 14 variables ranged from 0 to 2.7 %. In total, 3,938 out of 74,664 (5.2 %) were incomplete. We used multiple imputations to create and analyze 10 imputed datasets. Incomplete variables were imputed under fully conditional speculation. Missing data were estimated with multiple logistic regressions applied separately to each imputed dataset. These estimates and their standard errors were combined. For comparison, we also performed this analysis on a subset of complete cases, which did not produce material differences.

We examined the association between the perception of the risks of radiation exposure and behavior change among those who began smoking, compared with those who remained non-smokers, after the disaster. Additionally, we examined the association between the recognition of the risk of radiation exposure and behavior change among those who quit smoking, compared with those who continued smoking, after the disaster. Age and sex-adjusted odds ratios (ORs) and 95 % confidence intervals (CIs) for the changes in smoking status were calculated using logistic regression analysis. In a multivariable-adjusted analysis, the model was adjusted for the following variables: age, sex, physical exercise, education attainment, medical histories of hypertension, diabetes, hyperlipidemia, stroke, ischemic heart disease, cancer, and mental illness. The same analysis was conducted for drinking status. We classified age and sex to perform a subgroup analysis. P values were obtained using a two-tailed test, and p < 0.05 was regarded as statistically significant. We used SAS version 9.4 (SAS Institute Inc, Cary, NC) for all statistical analyses.

The study was conducted according to the guidelines of the Declaration of Helsinki, and approved by the Ethics Review Committees of Fukushima Medical University (Number 1316).

## 4 Results

The proportion of current smokers among the 74,667 participants decreased from 28.2 % to 23.0 % (male 35.9 % and female 11.7 %) after the disaster. Furthermore, 832 participants started smoking and 4,728 quit smoking after the disaster. The proportion of current drinkers among the 77,406 participants increased from 45.6 % to 47.0 % (male 67.0 % and female 29.8 %) after the disaster; 3,961 participants started drinking and 2,861 quit drinking after the disaster.

Table 1 shows the participants’ baseline characteristics in accordance with the perception of radiation exposure risk. Compared with participants who perceived the risk of negative health outcomes developing because of radiation exposure as “unlikely,” those who perceived the risk as “very likely” tended to be female, young, and have mental illness. In contrast, those who reported higher educational attainment and greater physical activity levels tended to perceive the radiation-related risk as “unlikely.”

As shown in Table 2, the adjusted ORs (95 % CIs) for starting smoking among participants who perceived radiation exposure risks as “unlikely,” “likely” or “very likely” in reference to “very unlikely” were 0.96 (0.78–1.18), 1.17 (0.95–1.45), and 1.69 (1.39–2.06), respectively (p for trend = 0.01). The corresponding ORs for quitting smoking were 0.90 (0.82–0.98), 0.81 (0.73–0.90), and 0.75 (0.68–0.83), respectively (p for trend = <0.01). There was a significant interaction between the perceptions of radiation exposure risk and sex (p = 0.038), but not age (p = 0.339), for starting smoking. When comparing the perceptions of radiation exposure risk, “very likely” and “very unlikely,” the ORs for starting smoking after the disaster were highest among 20–49-year-olds (OR: 2.12 [1.61–2.79]) and females (OR: 2.03 [1.46–2.82]) (Table S1).

**Table 2:** Multivariable odds ratios for starting smoking (n = 53,620) and quitting smoking (n = 21,047) after the Fukushima disaster, according to the perception of radiation exposure risk.

		Perception of radiation exposure risk			
		Very unlikely	Unlikely	Likely	Very likely
New smokers after the disaster among non smokers at the time of disaster (n = 53,620)					
	N	13,291	16,088	12,007	12,234
	Start smoking, n(%)	171 (1.3)	200 (1.2)	187 (1.6)	274 (2.2)
	OR (univariate)	ref	0.94 (0.77–1.16)	1.16 (0.94–1.43)	1.68 (1.38–2.04)
	OR(multi-variate)*	ref	0.96 (0.78–1.18)	1.17 (0.95–1.45)	1.69 (1.39–2.06)
New quitters of smoking after the disaster among smokers at the time of disaster (n = 21,047)					
	N	5021	6202	4538	5286
	Quit smoking, n(%)	1318 (26.2)	1410 (22.7)	950 (20.9)	1050 (19.9)
	OR (univariate)		0.83 (0.76–0.90)	0.74 (0.68–0.82)	0.70 (0.64–0.76)
	OR (multi-variate)*	ref	0.90 (0.82–0.98)	0.81 (0.73–0.90)	0.75 (0.68–0.83)
OR: odds ratio					

*Adjusted for age, sex, exercise, education, drinking status, experience of radiation accident, and medical history of hypertension, diabetes, hyperlipidemia, cancer, stroke, heart disease, and mental illness.

The multivariable ORs (95 % CIs) for starting drinking after the disaster among participants who perceived radiation exposure risks as “unlikely,” “likely,” or “very likely” in reference to “very unlikely” were 1.05 (0.95–1.16), 1.17 (1.06–01.30) and 1.38 (1.25–1.52), respectively (p for trend = <0.01). The corresponding ORs (95 % CIs) for quitting drinking were 1.07 (0.96–1.19), 1.05 (0.94–1.18) and 1.12 (1.00–1.25), respectively (p for trend = 0.27) (Table 3). There was a significant interaction between the perceptions of radiation exposure risk and sex (p = 0.019), and age (p < 0.001) for stopping drinking. The number of people who stopped drinking after the disaster was significantly higher among those in the young age group (20–49) and the female population who perceived more radiation exposure risk (Table S2).

**Table 3:** Multivariable odds ratios for starting drinking (n = 42,133) and stopping drinking (n = 35,273) after the Fukushima disaster, according to the perception of radiation exposure risk.

		Perception of radiation exposure risk			
		Very unlikely	Unlikely	Likely	Very likely
New drinkers after the disaster among participants who did not drink at the time of disaster (n = 42,133)					
	N	9935	12,483	9540	10,175
	n	787	1094	926	1154
	Start drinking, n(%)	787 (7.9)	1094 (8.8)	926 (9.7)	1154 (11.3)
	OR (univariate)	ref	1.06 (0.96–1.17)	1.20 (1.08–1.33)	1.43 (1.30–1.58)
	OR(multi-variate)*	ref	1.05 (0.95–1.16)	1.17 (1.06–1.30)	1.38 (1.25–1.52)
New quitters of drinking after the disaster among participants who drunk at the time of disaster (n = 35,273)					
	N	8934	10,577	7699	8063
	Stop drinking, n(%)	621 (7.0)	849 (8.0)	658 (8.5)	733 (9.1)
	OR (univariate)	ref	1.07 (0.96–1.20)	1.10 (0.98–1.23)	1.19 (1.06–1.33)
	OR(multi-variate)*	ref	1.07 (0.96–1.19)	1.05 (0.94–1.18)	1.12 (1.00–1.25)
OR: odds ratio					

*adjusted for age, sex, exercise, education, smoking status, experience of radiation accident, and medical history of hypertension, diabetes, hyperlipidemia, cancer, stroke, heart disease, and mental illness.

## 5 Discussion

The results of this study showed that the perception that radiation exposure increases the risk for developing cancer was positively associated with the likelihood of starting smoking and of starting alcohol-drinking among evacuees after the Great East Japan Earthquake in Fukushima. Moreover, this perception was inversely associated with the likelihood of quitting smoking following the disaster. The effect of radiation exposure is still vigorously discussed and definite conclusion may not be achieved. However, these behavior changes could potentially introduce higher risks than the radiation exposure itself. Future research should quantitatively assess those people who perceived the risk of radiation exposure and changed their health-related behavior; the high-risk group could thus be identified and proactive measures could be taken.

Previous studies have shown that the proportion of smokers tends to increase after a major disaster. For example, among people who suffered from the terrorist attacks in Manhattan on 11 September 2001, smokers increased from 22.6 % during the prior week to 23.4 % 4–7 weeks after the tragedy (Vlahov et al., 2002); and among people who experienced Hurricane Katrina; smokers increased from 34.3 % before to 52.5 % after the hurricane (Flory et al., 2009). After the Canterbury Earthquake in New Zealand; 24 % of ex-smokers relapsed (Erskine et al., 2013). Previous studies have explained that the increase in tobacco use was because people used tobacco to relax and to cope with stressful situations. Therefore; stress can be viewed as a mediator between tobacco use and the damage caused by the disaster. In our study the total number of smokers decreased from 28.2 % to 23.0 %. This decrease in tobacco use following the disaster was partly attributable to decreased access to tobacco products (Nakano et al., 2018). The earthquake disrupted railway and road transportation networks; and damaged tobacco plantations, which ceased tobacco production. The total tobacco volume between April and September 2011 decreased by 20.4 % compared with the same period in the previous year (JTI, 2011).

A previous cross-sectional study with 59,807 residents (56 % female) from Fukushima reported that poor mental health after the disaster was associated with the perception of radiation exposure risks (Suzuki et al., 2015). The research suggested that poor mental health arose from an incorrect understanding of the health effects of radiation. Therefore, people who perceived greater risk in radiation exposure may have had an incorrect understanding of the health effects of radiation exposure. In the case of Fukushima, the amount of radiation exposure was and is reportedly limited, while the risk associated with smoking is certain; tobacco is accountable for one in ten deaths worldwide (Reitsma et al., 2017). Thus, it is surely better for an individual not to smoke when they perceive radiation exposure as a risk and are afraid of developing cancer in the future. In addition, smoking does not reduce stress as is generally expected (Parrott, 1999). In the subgroup analysis, young and female people had an even higher probability of starting smoking after the disaster when they perceived more risk in radiation exposure. These groups would benefit from targeted information about health risks. Furthermore, the effects of radiation exposure should be continuously measured and disclosed to the population.

The percentage of evacuees who consumed alcohol increased slightly from 45.6 % to 47.0 %, and stronger perceptions of risk in radiation exposure was associated with starting drinking. Many previous studies have reported increased alcohol consumption among victims in response to major disasters, such as hurricanes, flooding, jet crashes, and terrorist bombings, although there is not a consensus about the association between traumatic events and alcohol consumption (Keyes et al., 2011). The perception of radiation exposure risk among evacuees in Fukushima Prefecture tended to include an aspect of psychological distress (Suzuki et al., 2015). Thus, people in Fukushima who perceived a risk in radiation exposure were more likely to start drinking after the disaster to cope with the distress they experienced during the evacuation process.

Interestingly, people who perceived the radiation exposure risk as very likely were significantly more likely to stop drinking compared with those who perceived the risk as very unlikely. This association was only significant among females and the group aged 20 to 49. Thus, it is possible that these people of childbearing age were more aware of their health after the disaster. Therefore, in those groups, the perception of radiation exposure changed drinking behavior in the opposite direction: those who drank stopped drinking, and those who did not drink started drinking.

Several limitations in this study must be addressed. First, we investigated smoking status before and after the earthquake using a self-reported questionnaire that was disseminated after the disaster. Although the question was simple, the retrospective design may have led to recall bias. Second, we did not assess the number of smoked cigarettes among current smokers and therefore we did not examine dose–response relationships. Third, as described in the Discussion, stress can be a mediator between perceived risk of developing cancer from radiation and behavior change, but we did not ask about the degree of stress in the survey. The association between stress levels and perceived risk of developing cancer should be further assessed.

## 6 Conclusion

People in Fukushima following the 2011 tsunami and nuclear plant disaster who perceived that radiation exposure posed risks of developing cancer in fact had higher odds of starting smoking and drinking, both of which potentially bring higher cancer risks than the level of radiation exposure experienced by the studied population. Thus, education regarding the health risks of smoking and alcohol consumption should be improved.

## CRediT authorship contribution statement

**Tomohiko Ukai:** Conceptualization, Methodology, Formal analysis, Writing – original draft, Writing – review & editing. **Takahiro Tabuchi:** Conceptualization, Methodology, Writing – review & editing, Supervision. **Tetsuya Ohira:** Conceptualization, Methodology, Supervision. **Hironori Nakano:** Conceptualization, Supervision. **Masaharu Maeda:** Conceptualization, Writing – review & editing, Supervision. **Hirooki Yabe:** Conceptualization, Supervision. **Atsushi Takahashi:** Conceptualization, Supervision. **Seiji Yasumura:** Conceptualization, Writing – review & editing, Supervision. **Hiroyasu Iso:** Methodology, Formal analysis, Writing – review & editing, Supervision. **Kenji Kamiya:** Conceptualization, Writing – review & editing, Supervision.

## Declaration of Competing Interest

The authors declare that they have no known competing financial interests or personal relationships that could have appeared to influence the work reported in this paper.

## Data Availability

The authors do not have permission to share data.
