# Supplementary material for: Associations between the perception of risk in radiation exposure and changes in smoking and drinking status after a disaster: The Fukushima Health Management Survey
Source: Prev Med Rep. 2022 Nov 14;30:102054. doi: 10.1016/j.pmedr.2022.102054 (PMC9747623; doi:10.1016/j.pmedr.2022.102054)
Supplement: Supplementary data 1 [file mmc1.docx]

| Table S1. Multivariable odds ratios for starting smoking and quitting smoking after the Fukushima disaster, according to the perception of radiation exposure risk classified by age and sex. | | | | | |
| --- | --- | --- | --- | --- | --- |
|  |  | Perception of radiation exposure risk | | |  |
|  |  | Very unlikely | Unlikely | Likely | Very likely |
| New smokers after the disaster | | |  |  |  |
| Age (20–49) | N | 3467 | 5173 | 4142 | 3832 |
|  | n (%) | 80 (2.3) | 124 (2.4) | 112 (2.7) | 171 (4.5) |
|  | OR* | ref | 1.17 (0.88–1.56) | 1.30 (0.96–1.74) | 2.12 (1.61–2.79) |
| Age (50–64) | N | 4191 | 5271 | 3579 | 3805 |
|  | n (%) | 53 (1.3) | 44 (0.8) | 40 (1.1) | 60 (1.6) |
|  | OR* | ref | 0.74 (0.49–1.11) | 1.01 (0.67–1.54) | 1.33 (0.91–1.94) |
| Age (≥65) | N | 5633 | 5644 | 4286 | 4597 |
|  | n (%) | 38 (0.7) | 32 (0.6) | 35 (0.8) | 43 (0.9) |
|  | OR* | ref | 0.86 (0.54–1.39) | 1.22 (0.77–1.95) | 1.35 (0.86–2.11) |
| Sex (female) | N | 7650 | 10384 | 8091 | 8062 |
|  | n (%) | 51 (0.7) | 69 (0.7) | 75 (0.9) | 130 (1.6) |
|  | OR* | ref | 0.90 (0.62–1.29) | 1.16 (0.81–1.66) | 2.03 (1.46–2.82) |
| Sex (male) | N | 5641 | 5704 | 3916 | 4172 |
|  | n (%) | 120 (2.1) | 131 (2.3) | 112 (2.9) | 144 (3.5) |
|  | OR* |  | 1.02 (0.80–1.32) | 1.23 (0.95–1.61) | 1.50 (1.16–1.92) |
|  |  |  |  |  |  |
| New quitters of smoking after the disaster | | |  |  |  |
| Age (20–49) | N | 1908 | 3046 | 2529 | 2935 |
|  | n (%) | 361 (18.9) | 575 (18.9) | 437 (17.3) | 471 (16.0) |
|  | OR* | ref | 0.97 (0.83–1.13) | 0.84 (0.72–0.99) | 0.76 (0.65–0.89) |
| Age (50–64) | N | 1917 | 2067 | 1297 | 1526 |
|  | n | 488 | 440 | 263 | 294 |
|  | n (%) | 488 (25.5) | 440 (21.3) | 263 (20.3) | 294 (19.3) |
|  | OR* | ref | 0.83 (0.71–0.97) | 0.78 (0.65–0.92) | 0.72 (0.60–0.85) |
| Age (≥65) | N | 1196 | 1089 | 712 | 825 |
|  | n (%) | 469 (39.2) | 395 (36.3) | 250 (35.1) | 285 (34.5) |
|  | OR* | ref | 0.89 (0.74–1.05) | 0.84 (0.68–1.02) | 0.79 (0.65–0.96) |
| Sex (female) | N | 936 | 1594 | 1433 | 1707 |
|  | n (%) | 245 (26.2) | 387 (24.3) | 327 (22.8) | 375 (22.0) |
|  | OR* | ref | 0.92 (0.76–1.12) | 0.85 (0.70–1.04) | 0.82 (0.68–1.00) |
| Sex (male) | N | 4085 | 4608 | 3105 | 3579 |
|  | n (%) | 1073 (26.3) | 1023 (22.2) | 623 (20.1) | 675 (18.9) |
|  | OR* | ref | 0.88 (0.80–0.98) | 0.79 (0.71–0.89) | 0.72 (0.64–0.81) |
| OR: odds ratio |  |  |  |  |  |
| *adjusted for age, sex, exercise, education, drinking status, experience of radiation accident, and medical history of hypertension, diabetes, hyperlipidemia, cancer, stroke, heart disease, and mental illness | | | | | |

| Table S2. Multivariable odds ratios for starting drinking and stopping drinking after the Fukushima disaster, according to the perception of radiation exposure risk classified by age and sex. | | | | | |
| --- | --- | --- | --- | --- | --- |
|  |  | Perception of radiation exposure risk | | |  |
|  |  | Very unlikely | Unlikely | Likely | Very likely |
| New drinkers after the disaster | | |  |  |  |
| Age (20–49) | N | 2660 | 4391 | 3529 | 3613 |
|  | n(%) | 335 (12.6) | 548 (12.5) | 484 (13.7) | 600 (16.6) |
|  | OR* | ref | 0.99 (0.86–1.15) | 1.12 (0.96–1.30) | 1.35 (1.17–1.56) |
| Age (50–64) | N | 2840 | 3715 | 2568 | 2780 |
|  | n(%) | 217 (7.6) | 301 (8.1) | 235 (9.2) | 292 (10.5) |
|  | OR* | ref | 1.13 (0.94–1.35) | 1.28 (1.05–1.56) | 1.45 (1.20–1.75) |
| Age (≥65) | N | 4435 | 4377 | 3443 | 3782 |
|  | n(%) | 235 (5.3) | 245 (5.6) | 207 (6.0) | 262 (6.9) |
|  | OR* | ref | 1.06 (0.88–1.28) | 1.17 (0.96–1.42) | 1.37 (1.14–1.65) |
| Sex (female) | N | 6580 | 8954 | 7053 | 7337 |
|  | n(%) | 408 (6.2) | 647 (7.2) | 562 (8.0) | 673 (9.2) |
|  | OR* | ref | 1.04 (0.92–1.19) | 1.14 (1.00–1.31) | 1.33 (1.17–1.52) |
| Sex (male) | N | 3355 | 3529 | 2487 | 2838 |
|  | n(%) | 379 (11.3) | 447 (12.7) | 364 (14.6) | 481 (16.9) |
|  | OR* |  | 1.08 (0.93–1.25) | 1.27 (1.08–1.48) | 1.50 (1.29–1.74) |
|  |  |  |  |  |  |
| New quitters of drinking after the disaster | | |  |  |  |
| Age (20–49) | N | 2800 | 4001 | 3276 | 3338 |
|  | n(%) | 202 (7.2) | 434 (10.8) | 358 (10.9) | 362 (10.8) |
|  | OR* | ref | 1.39 (1.16–1.66) | 1.34 (1.11–1.62) | 1.35 (1.12–1.62) |
| Age (50–64) | N | 3474 | 3862 | 2506 | 2740 |
|  | n(%) | 149 (4.3) | 193 (5.0) | 121 (4.8) | 164 (6.0) |
|  | OR* | ref | 1.12 (0.90–1.40) | 0.95 (0.74–1.22) | 1.19 (0.94–1.50) |
| Age (≥65) | N | 2660 | 2714 | 1917 | 1985 |
|  | n(%) | 270 (10.2) | 222 (8.2) | 179 (9.3) | 207 (10.4) |
|  | OR* | ref | 0.78 (0.64–0.94) | 0.85 (0.69–1.04) | 0.92 (0.75–1.12) |
| Sex (female) | N | 2344 | 3554 | 2916 | 2953 |
|  | n(%) | 270 (11.5) | 488 (13.7) | 424 (14.5) | 434 (14.7) |
|  | OR* | ref | 1.19 (1.01–1.40) | 1.25 (1.05–1.47) | 1.25 (1.06–1.48) |
| Sex (male) | N | 6590 | 7023 | 4783 | 5110 |
|  | n(%) | 351 (5.3) | 361 (5.1) | 234 (4.9) | 299 (5.9) |
| OR: odds ratio |  |  |  |  |  |
| *adjusted for age, sex, exercise, education, smoking status, experience of radiation accident, and medical history of hypertension, diabetes, hyperlipidemia, cancer, stroke, heart disease, and mental illness | | | | | |
